# Efficacy of Second-Line Treatments After Atezolizumab and Bevacizumab in Advanced Hepatocellular Carcinoma and Related Prognostic Factors: A Multicenter Study by the Turkish Oncology Group (TOG)

**DOI:** 10.5152/tjg.2025.24784

**Published:** 2025-04-07

**Authors:** Nargiz Majidova, Sendag Yaslıkaya, Maral Martin Mıldanoglu, Alper Coskun, Duygu Ercan Uzundal, Taha Koray Sahin, Arif Akyildiz, Sinem Akbas, Ufuk Camanlı, Elif Sahin, Huseyin Atacan, Ismail Bayrakcı, Teoman Sakalar, Ceren Mordag Cicek, Damla Gunenc, Nurullah İlhan, Olcun Umit Unal, Ozkan Alan, Buket Hamitoglu, Esra Ozen Engin, Nadiye Sever, Ali Kaan Guren, Ahmet Unsal, Murat Araz, Bulent Erdogan, Musa Barıs Aykan, Fatih Selcukbiricik, Deniz Can Guven, Nuriye Ozdemir, Ahmet Bilgehan Sahin, Ahmet Bilici, Ismail Oguz Kara, Suayip Yalcın, Osman Kostek

**Affiliations:** 1 Division of Medical Oncology, VM Medical Park Maltepe Hospital, İstanbul, Türkiye; 2 Division of Medical Oncology, Çukurova University School of Medicine, Adana, Türkiye; 3 Division of Medical Oncology, Medipol University Hospital, İstanbul, Türkiye; 4 Division of Medical Oncology, Uludağ University Faculty of Medicine, Bursa, Türkiye; 5 Division of Medical Oncology, Gazi University, Ankara, Türkiye; 6 Division of Medical Oncology, Hacettepe University Cancer Institute, Ankara, Türkiye; 7 Division of Medical Oncology, Koç University Hospital, İstanbul, Türkiye; 8 Division of Medical Oncology, İzmir Bozyaka Training and Research Hospital, İzmir, Türkiye; 9 Division of Medical Oncology, Kocaeli City Hospital, Kocaeli, Türkiye; 10 Division of Medical Oncology, Gülhane Training and Research Hospital, Ankara, Türkiye; 11 Division of Medical Oncology, Trakya University, İstanbul, Türkiye; 12 Division of Medical Oncology, Kahramanmaraş Necip Fazıl City Hospital, Kahramanmaraş, Türkiye; 13 Division of Medical Oncology, Pamukkale University Hospital, Denizli, Türkiye; 14 Division of Tulay Aktas Oncology, Ege University, İzmir, Türkiye; 15 Division of Medical Oncology, İstanbul Sancaktepe Şehit Prof. Dr. İlhan Varank Training and Research Hospital, İstanbul, Türkiye; 16 Division of Medical Oncology, Tepecik Training and Research Hospital, İzmir, Türkiye; 17 Division of Medical Oncology, Eylül University Faculty of Medicine, İzmir, Türkiye; 18 Division of Medical Oncology, Sakarya University School of Medicine, Sakarya, Türkiye; 19 Division of Medical Oncology, Marmara University School of Medicine, İstanbul, Türkiye; 20 Division of Medical Oncology, Gümüşhane State Hospital, Gümüşhane, Türkiye; 21 Division of Medical Oncology, Necmettin Erbakan University Meram Medical Faculty Hospital, Konya, Türkiye

**Keywords:** Atezolizumab, bevacizumab, cabozantinib, hepatocellular carcinoma, overall survival, progression-free survival, regorafenib, second-line treatment, sorafenib

## Abstract

**Background/Aims:**

The treatment of hepatocellular carcinoma (HCC), which accounts for 90% of all liver cancers, is highly varied. The use of second-line treatments following progression on first-line atezolizumab and bevacizumab (Atez/Bev) for advanced HCC remains controversial. The aim of this study was to analyze the real-world clinical results of second-line treatments in progression after Atez/Bev and to determine the factors affecting prognosis.

**Materials and Methods:**

Fifty-eight patients treated with second-line sorafenib, regorafenib, and cabozantinib for progression after first-line Atez/Bev for advanced/metastatic HCC from 20 centers in Türkiye between October 2020 and June 2024 were retrospectively analyzed. Responses were evaluated by Response criteria, specifically Response Evaluation Criteria in Solid Tumors (RECIST v1.1) criteria. Median overall survival (OS) and progression-free survival (PFS) were computed with the Kaplan–Meier method. The Cox regression model was utilized to analyze multivariate analyses.

**Results:**

About 82.8% of the patients were male and the median age of the whole group was 62 (range, 18-78) years. All patients progressed after first-line Atez/Bev and were given second-line treatment. The most commonly used second-line treatment option was sorafenib (70.7%), followed by regorafenib (12.1%) and cabozantinib (10.3%). Both median PFS (4.1 months) and median OS (7.8 months) were longer in patients treated with sorafenib compared to other treatments. In univariate analyses, Child–Pugh score B, high alpha-fetoprotein (AFP) levels (>200 ng/mL), extrahepatic spread, and Prognostic Nutritional Index (PNI) < 47.6 substantially raised the risk of overall mortality. Multivariate analysis showed that extrahepatic spread (HR (Hazard ratio): 0.41, *P* = .012), PNI level (HR: 0.24, *P* = .005), and AFP level (HR:1.97, *P* = .049) were independent predictors of OS.

**Conclusion:**

Although second-line therapies after Atez/Bev show different degrees of efficacy, survival rates are consistent with the literature. Extrahepatic spread, AFP level, and PNI level are the main prognostic factors. In light of this information, personalized treatment strategies may improve outcomes for this challenging patient group.

**Main Points**

- Treatment approaches for advanced hepatocellular carcinoma following progression with first-line atezolizumab and bevacizumab.
- Survival outcomes of second-line therapies in advanced hepatocellular carcinoma.
- Prognostic indicators of survival outcomes in advanced HCC; such as extrahepatic spread, alpha-fetoprotein (AFP) levels and Prognostic Nutritional Index (PNI).

## Introduction

Hepatocellular carcinoma (HCC), the most widespread cancer of the liver, poses a major global health problem with high prevalence and mortality rates.^1^,^2^ Although curative treatments are effective in appropriate patients, these treatments are inadequate for the majority of patients.^3^ First-line treatment in HCC is IO-IO(IO- immunotherapy) combination or IO-TKI (Immunotherapy-Tyrosine Kinase inhibitors) combination,^4^^-^ ^8^ but data evaluating treatment options for second-line treatment are limited. The combination of atezolizumab, an anti-PD-L1 antibody, and bevacizumab, an anti-VEGF monoclonal antibody, is the first-line standard of care for unresectable or metastatic HCC.^9^ However, despite this treatment, progression develops in a significant number of patients and therefore second-line therapies are needed. In this regard, second-line therapies frequently involve TKIs, including sorafenib, regorafenib, cabozantinib, and lenvatinib. Each drug targets multiple pathways involved in angiogenesis, tumor growth, and metastasis. However, studies on the efficacy of these drugs post-Atez/Bev are limited and remain an ongoing area of real-world research. In this difficult-to-manage patient group, it is critical to identify the most optimal treatment options in the second-line and to investigate the factors that affect treatment outcomes.

In recent years, prognostic markers reflecting the general health status and immunologic response of cancer patients have become increasingly important in treatment planning.^10^^-^ ^12^ The Prognostic Nutritional Index (PNI) has emerged as an effective tool for predicting the prognosis of cancer patients by combining nutritional status and immune function.

In this study, the aim was to assess the effectiveness of second-line therapies in progression after Atez/Bev in patients with locally refractory or metastatic HCC and to analyze treatment responses, survival outcomes, and potential prognostic factors to provide insights to optimize second-line management.

## Materials and Methods

### Study Design and Participants

This retrospective multicenter study (20 centers) included patients with HCC (histopathologic or radiologic) refractory to local therapies or metastatic at the time of diagnosis who had progressed after first-line Atez/Bev and received second-line treatment between 2020 and 2024. In Türkiye, the number of patients receiving second-line treatment was low, as access to first-line Atez/Bev treatment is difficult due to payment conditions. Therefore, all patients whose data could be accessed were evaluated, not just a homogeneous group. All patients had pure HCC.

Demographic characteristics, laboratory parameters before second-line treatment (alpha-fetoprotein (AFP) level, total bilirubin, albumin, lymphocyte count, etiology of liver disease, presence of cirrhosis and extrahepatic spread, transarterial chemoembolization (TACE) or transarterial radioembolization (TARE) and current treatments, and survival time were recorded from files or electronically.

Furthermore, PNI scores were calculated as serum albumin (g/L) + 5 × total lymphocyte count (10^9^/L).

Progression-free survival was defined as the time from the start of second-line treatment to disease progression or death. Overall survival (OS) was defined as the time from the start of second-line treatment to death from any cause.

### Treatment Characteristics

Patients received one of the following second-line therapies according to the clinician’s choice: sorafenib, regorafenib, cabozantinib, or other systemic agents (chemotherapy, lenvatinib, or ramucirumab). Response to treatment was evaluated according to RECIST version 1.1 criteria. The first response evaluation was performed radiologically 3 months after the start of treatment. The highest objective response rate (ORR) was classified as complete response (CR), partial response (PR), stable disease (SD), or progressive disease (PD). Objective response rate was defined as the sum of CR + PR; disease control rate (DCR) was defined as the sum of CR + PR + SD.

### Statistical Analysis

Descriptive statistics were used to summarize baseline characteristics. Kaplan–Meier survival curves were constructed to evaluate Progression-free survival (PFS) and OS, and log-rank tests were used to compare survival distributions between treatment groups. The chi-square test was used to compare independent categorical variables. According to receiver operating characteristic (ROC) curve analysis, the optimum cut-off value for PNI was determined as 47.6. Statistical analyses were performed using IBM SPSS Statistics software (version 23) (IBM SPSS Corp.; Armonk, NY, USA), and a value of *P* < .05 was accepted to indicate statistical significance.

This study was conducted in accordance with the principles outlined in the Declaration of Helsinki. Approval was first given as a single center by the ethics committee of Marmara University. Subsequently, multi-center approval was granted due to an insufficient number of patients, upon submitting a petition (approval number 09.2023.1552; date: October 4, 2024).

## Results

### Baseline Characteristics

The median age of the 58 patients included in the study was 62 years (range 18-78) and the majority of the patients were male (82.8%). Child–Pugh score was 5 in 60.3% of patients, and 48.3% had BCLC (Barcelona Clinic Liver Cancer Stages) stage B, while 51.7% had stage C. Hepatitis B virus (HBV) was the etiology of cancer in 41.4%. Cirrhosis was present in 46.6% of patients. Extrahepatic spread was present in 44.8% of cases: lung (20.7%), bone (10.3%), and other sites (13.8%), respectively. All patients progressed, and a second line of treatment was initiated. As second-line treatment, 41 patients (70.7%) received sorafenib, 6 (10.3%) cabozantinib, 7 (12.1%) received regorafenib, and 4 patients received other therapies (capecitabine, gemcitabine + oxaliplatin, lenvatinib, ramucirumab, respectively) (Table 1).

### Treatment Responses

The best responses with second-line therapies were observed in 18% of patients treated with sorafenib. The DCR was highest in the sorafenib group (59%), followed by cabozantinib (50%) and regorafenib (28%) (Table 2).

Patients treated with sorafenib were re-evaluated according to achieved disease control. Age, gender, etiology, cirrhosis status, ECOG-PS, and PNI were not significantly associated with disease control. However, patients with higher AFP levels (>200 ng/mL) were less likely to achieve DCR (76% vs. 33%, *P* = .006), and those with Child–Pugh score B had significantly lower DCR (21% vs. 53%, *P* = .03) (Table 3).

### Survival Analysis

During a median follow-up of 20.4 (95% CI 17.0-23.7) months, HCC progressed in 49 (84.5%) patients and 40 (69%) patients died from it. Median PFS in the whole group was 4.1 (95% CI 2.4-5.7) months. Median OS in the whole group was 6.5 (95% CI 2.7-10.2) months. The median PFS was 4.1 (95% CI 2.4-5.8) months for sorafenib, 2.2 (95% CI 1.1-3.2) months for regorafenib, and 2.8 (95% CI 1.2-3.5) months for cabozantinib. The median OS was 7.8 (95% CI 2.9-12.7) months for sorafenib, 2.3 (95% CI 1.7-2.9) months for regorafenib, and 3.7 (95% CI 2.9-6.0) months for cabozantinib. Survival analysis of the remaining patients who had different treatments is presented as a case series. For a patient receiving lenvatinib, PFS was 4.4 months, and OS was 23.7 months. For a patient receiving ramucirumab, both PFS and OS were 1 month. With chemotherapy, PFS was 2.7 months, and OS was 6.5 months. In case series of patients, the best response was PD, while the best response with lenvatinib was SD.

**Figure 1.**
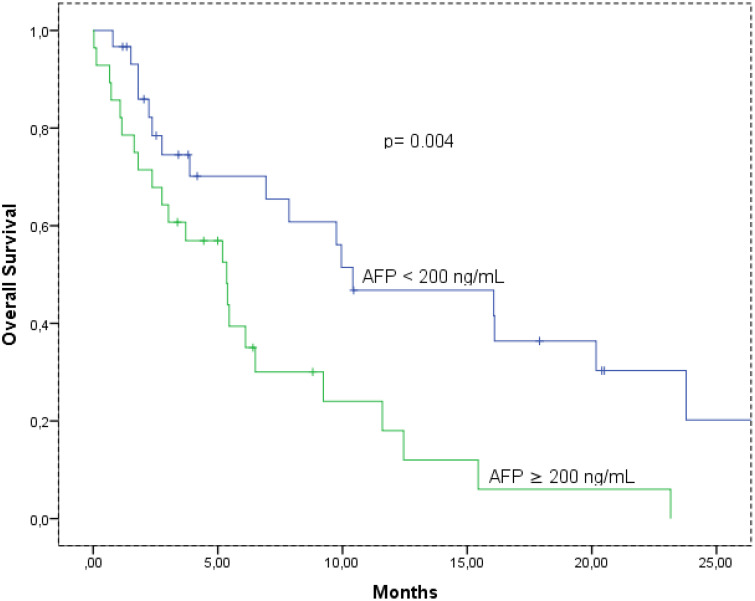
Overal survival according to alpha-fetoprotein.

**Figure 2.**
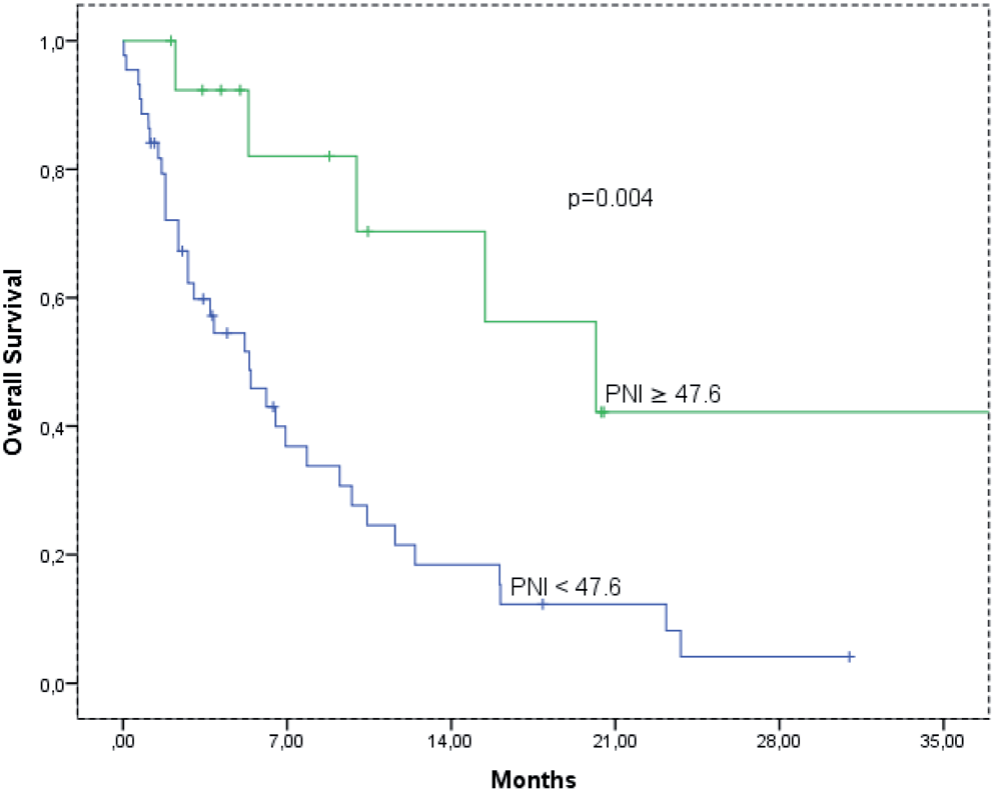
Overal survival according to prognostic nutritional index cut-off. mOS, median overall survival; PNI, prognostic nutritional index.

The median OS times according to AFP level are shown in Figure 1. The median OS was 10.4 (95% CI: 1.5-19.2), and 5.3 (95% CI: 2.9-7.8) months, in the AFP low and high group, respectively (*P* = .004). Also, the median OS was 20.1 (95% CI: 8.4-31.8), and 20.1 (95% CI: 8.4-31.8) months, in the PNI ≥ 47.6 and PNI < 47.6 group, respectively (*P* = .004) (Figure 2).

**Figure 3.**
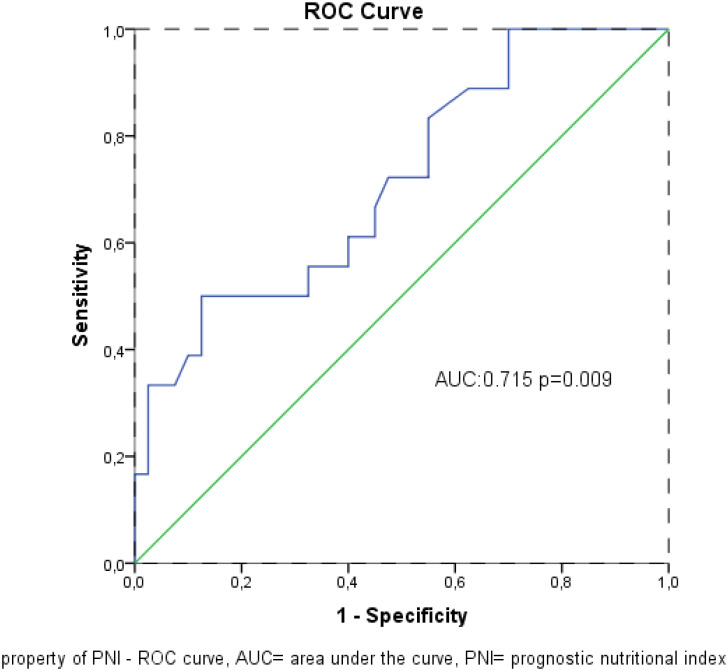
Mortality predictive property of PNI – ROC curve. AUC, area under the curve; PNI, prognostic nutritional index.

Receiver operating characteristic analysis revealed that PNI was a significant prognostic factor for mortality (Area under the curve: 0.715; 95% CI: 0.572-0.857; *P* = .009; Figure 3). When the cut-off value was > 47.6, the specificity and sensitivity of the test were 76.7% and 65.7%, respectively.

Univariate analysis for OS identified AFP ≥ 200 ng/mL (HR: 2.54, *P* = .006), Child–Pugh score B (HR: 2.91, *P* = .002), extrahepatic spread (HR: 0.51, *P* = .04) and PNI (HR: 0.27, *P* = .0008) as significant predictors of OS. In multivariate analysis, extrahepatic spread (HR: 0.41, *P* = .012), PNI level (HR: 0.24, *P* = .005), and AFP level (HR:1.97, *P* = .049) were independent predictors of OS (Table 4).

## Discussion

Although first-line therapies in the treatment of locally advanced HCC are widely available in the literature, second-line therapies are still controversial. In this study, the drug efficacy and prognostic factors with second-line therapies after first-line Atez/Bev in patients with HCC refractory to local therapies or metastatic at diagnosis were evaluated. The results highlighted variable outcomes between different second-line therapies, particularly sorafenib, regorafenib, and cabozantinib, and the key factors affecting DCR, PFS, and OS in this patient population were also assessed.

Sorafenib is among the most frequently used treatments, reflecting its established role in managing advanced HCC. Although the efficacy of other TKIs in progression after sorafenib in the first line is known, the efficacy of second-line therapies in progression after Atez/Bev in this line is still controversial, and there is insufficient data.^13^,^14^ The results show that sorafenib achieved a DCR of 59% and a median PFS of 4.1 months, which is comparable to previous studies of its use in similar settings.^15^^-^^18^ Sorafenib remains an important option for the treatment of HCC and this has been supported by larger studies.

Regorafenib and cabozantinib remain a viable option for selected patients, despite the lower DCR (28% vs. 50%) and PFS (2.2 months vs. 2.8 months). These results suggest that sorafenib showed higher efficacy. However, the shorter PFS times in this study compared to other studies in the literature may be due to the small sample size and thus limited statistical power of the results.^19^,^20^

In addition, in this study, OS was longer with sorafenib compared to other treatment options. This suggests that sorafenib has a stronger effect on OS in HCC treatment and provides more benefit, especially in advanced patients. In the literature, the positive effect of sorafenib on survival times has also been confirmed in studies conducted particularly in patients with advanced-stage disease and metastatic status. These findings support the role and efficacy of sorafenib in the treatment of HCC and provide an important guide for treatment selection.^21^,^22^

In this study, only 1 patient received lenvatinib as second-line treatment. This patient had a PFS of 4.4 months and an OS of 23.7 months. In the literature, the efficacy of lenvatinib in HCC has been evaluated in a limited number of studies, especially in patients with progression after systemic therapy. Lenvatinib is a proven treatment option in the REFLECT trial, offering better PFS and OS times compared to sorafenib. However, the prolonged OS in this single case using lenvatinib after atezolizumab and bevacizumab highlights the potential efficacy of this treatment option.

The use of ramucirumab as a second-line treatment for HCC patients with AFP > 1000 ng/mL is an approach that has been shown to be effective for this subgroup in studies.^23^ However, progression-free survival (PFS) and OS of only 1 month suggest that the response to this treatment is very limited. Although ramucirumab has shown efficacy in patients with AFP > 1000 ng/mL, this group is generally associated with more aggressive disease. Therefore, other prognostic factors (e.g., performance status, Child–Pugh score) may significantly influence treatment outcomes. The limited efficacy of ramucirumab may reflect the heterogeneous nature of HCC and the need for a more detailed understanding of the association of elevated AFP with treatment response. These results may suggest that not only ramucirumab but also combined or different approaches should be considered in patients with high AFP levels.

It was noticed that the rate of HCC without cirrhosis in this cohort was higher than the rates typically reported in the literature. The reasons for this may be due to the population characteristics of this study. It should be considered that the etiology of HCC is different especially in Türkiye and chronic HBV is an important factor. Chronic HBV can lead to the development of HCC without cirrhosis. This may explain the high cirrhosis-free rate in this study. Some studies reported that HCC without cirrhosis has a more aggressive course in the Turkish population. In this study, genetic or environmental factors that may lead to a more aggressive clinical course in this population were not evaluated. However, it was observed that the results are consistent with previous findings and that this group shows a more aggressive course, which needs to be further investigated with larger-scale studies and biomarker analyses.^24^

In this study, 4 patients were Child–Pugh B. Treatment options are very limited for Child–Pugh B patients. Efficacy and safety data are generally lacking in clinical trials for this group. However, in the case of advanced disease, the need to provide treatment to patients before their clinical condition worsens may necessitate treatment for such patients. Although Child–Pugh B patients are known to have a poor prognosis, their overall performance status (e.g., ECOG 0-1) may be a supportive factor for treatment. Due to the lack of sufficient data in the literature on the efficacy of second-line therapies in Child–Pugh B patients, treatment in this patient group is often supported by “real-world” data. This is an individualized treatment decision made between the patient and the clinical team in light of limited data.

In this study, some prognostic factors were found to help overcome the difficulty in selecting second-line therapies. In patients on second-line sorafenib, higher AFP levels were significantly associated with lower DCR (76% vs. 33%, *P* = .006), which is consistent with studies in the literature that associate higher AFP levels with worse prognosis in HCC.^25^ Similarly, patients with Child–Pugh score B had significantly lower DCR compared to those with Child–Pugh score A, possibly due to impaired liver reserve. These findings are useful for treatment selection and prognosis prediction.

In terms of OS, extrahepatic spread, PNI level, and AFP level were independent predictors of survival in the multivariate analysis. Extrahepatic spread had a significantly worse prognosis (HR: 0.41; *P* = .012), indicating that metastasis is associated with survival outcomes in HCC. Moreover, AFP level was also a strong predictor of survival (HR: 1.97, *P* = .049). Low PNI levels (HR: 0.24, *P* = .005), i.e., low albumin levels, indicate malnutrition, which is an unfavorable prognosis in cancer. In this study, PNI was found to be a significant prognostic factor in both univariate and multivariate analyses. There is much evidence in the literature that PNI has been used to determine the prognosis of patients with various types of cancer, reflecting the relationship between nutritional status and immune function.^10^ The findings of the study suggest that low values of PNI are associated with poor prognosis and may be an important determinant in the treatment process. These results suggest that PNI should be evaluated more broadly in the treatment planning of HCC patients and its inclusion in clinical practice may improve the treatment response of patients. However, it is clear that more prospective studies are needed in this regard and the effect of PNI in combination with other prognostic factors should be examined in more detail. It should be emphasized that the use of PNI as a potential biomarker in second-line treatment applications in HCC may play an important role in individualizing treatment strategies.

An important strength of this study is that it provides detailed information on a specific geographical region with data from 20 different centers across Türkiye. Such comprehensive data provide an important contribution to understanding regional differences. Previous studies on unresectable HCC in Türkiye have provided valuable information on basic demographics, etiology distribution, and survival outcomes.^26^,^27^ In particular, the comparison of etiology distribution and survival outcomes may provide an important perspective on HCC management and treatment approaches in Türkiye. In this context, framing the study in relation to previous studies will strengthen the comprehensibility of the findings and their contribution to the general literature.

The results of this study provide valuable insights for selecting second-line therapies for progression after Atez/Bev in first-line HCC refractory to local therapies or metastatic cases. The findings are noteworthy as they indicate that the efficacy of sorafenib is superior to cabozantinib and regorafenib in terms of DCR, PFS, and OS outcomes. These findings are crucial for treatment decision-making in this challenging patient group, especially in light of the increased use of Atez/Bev. The findings suggest that patients may derive the most benefit from sorafenib, emphasizing its role as a viable option in subsequent treatment lines. Although less effective in terms of PFS and DCR, regorafenib and cabozantinib may also be considered, especially for patients with fewer treatment options. The results are supported by studies in the literature on second-line therapies after failure of atezolizumab and bevacizumab treatment.^28^^-^^33^ This study also highlighted the importance of prognostic factors such as PNI level, extrahepatic spread, and AFP level in the need for personalized treatment plans. Tailoring second-line therapies according to these variables can maximize patient outcomes and minimize the risk of side effects.

Although this study has several limitations. In particular, the limited sample size of other drugs compared with sorafenib reduces the generalizability of the results. In addition, the retrospective and multicenter study introduces possible biases regarding patient selection based on clinician selection. However, the fact that 20 centers from Türkiye have data reflects the treatment protocols and demographic data in the country. Larger-scale, prospective studies are needed to confirm the findings and to investigate the efficacy of other TKIs, such as sorafenib.

In conclusion, the findings demonstrated the efficacy of sorafenib as a second-line treatment for progression after Atez/Bev in patients with HCC refractory to local therapies or metastatic at diagnosis. In selected patients, cabozantinib, lenvatinib, and regorafenib may also be considered. In addition to these findings, factors such as PNI level, extrahepatic spread and AFP level also play an important role in determining prognosis. These findings demonstrated the importance of personalized treatment strategies in second-line therapy in HCC and may be the subject of future research to confirm the role of sorafenib in this respect.

## Figures and Tables

**Table 1.** Baseline Characteristics of Patients

Characteristics	n (%)
Age, Median (range), Years	62 (18-78)
Gender Female Male	10 (17.2) 48 (82.8)
ECOG-PS 0 1-2	24 (41.4) 34 (58.6)
Child–Pugh Score 5 6 7 8	35 (60.3) 19 (32.8) 3 (5.2) 1 (1.7)
BCLC Stage B C	28 (48.3) 30 (51.7)
Etiology Hepatits B Hepatitis C NASH Alcohol Others	24 (41.4) 5 (8.6) 5 (8.6) 2 (3.4) 22 (37.9)
Cirrhosis Yes No	27 (46.6) 31 (53.4)
Extrahepatic metastasis Lung Bone Others	12 (20.7) 6 (10.3) 8 (13.8)
Lesion size <5 cm >5 cm Unknown	13 (22.4) 37 (63.8) 8 (13.8)
Prior therapy TACE TARE	10 (17.2) 16 (27.6)
Histopathology Yes No	49 (84.5) 9 (15.5)
Second-line treatment option Sorafenib Cabozantinib Regorafenib Others**	41 (70.7) 6 (10.3) 7 (12.1) 4 (6.9)
AFP, median (range), ng/mL	160 (0.95-147314)
Total bilirubin, median (range), mg/dL	1.27 (0.2-68)
Albumin, median (range), g/dl	35 (16-49)
Lymphocyte, median (range), 10^3^/μL	1.1 (0.3-9.8)
INR, median (range)	1.1 (0.89-5.0)

AFP, alpha-fetoprotein; BCLC, Barcelona Clinic Liver Cancer; ECOG-PS, Eastern Cooperative Oncology Group-Performance Status; INR, international normalized ratio; NASH, nonalcoholic steatohepatitis; TACE, transarterial chemoembolization; TARE, transarterial radioembolization. **capecitabine; gemcitabine + oxaliplatine; lenvatinib; ramucirumab.

**Table 2.** Best Responses with Second-Line Treatments

Response	Second-Line Treatments, n/n (%)		
	Sorafenib	Regorafenib	Cabozantinib
Complete response (CR)	–	–	–
Partial response (PR)	7/41 (18)	1/7 (14)	2/6 (33)
Stable disease (SD)	17/41 (41)	1/7 (14)	1/6 (17)
Progressive disease (PD)	17/41 (41)	5/7 (72)	3/6 (50)
Objective response rate (CR+PR)	7/41 (18)	1/7 (14)	2/6 (33)
Disease control rate (CR+PR+SD)	24/41 (59)	2/7 (28)	3/6 (50)

**Table 3.** Comparison of Sorafenib-Treated Patients with and Without Disease Control

**Parameters**	**Disease Control, n (%)**		***P***
	**No, n = 17**	**Yes, n = 24**	
Age ≥ 65 years	7 (41)	10 (42)	.970
Male sex	14 (82)	21 (88)	.640
ECOG-PS ≥ 1	9 (53)	15 (63)	.540
Cirrhosis	8 (47)	8 (33)	.370
Viral hepatitis	8 (47)	13 (54)	.650
AFP, ng/mL ≥ 200	13 (76)	8 (33)	**.006**
Child–Pugh class B	9 (53)	5 (21)	**.030**
PNI ≥ 47.6	2 (18)	8 (33)	.113

Bold values indicate statistical significance.

AFP, alpha-fetoprotein; ECOG-PS, Eastern Cooperative Oncology Group-Performance Status; PNI, prognostic nutritional index.

**Table 4.** Univariate and Multivariate Analysis of Potential Prognostic Factors for Overall Survival with Sorafenib

Parameters	Univariate	*P*	Multivariate	*P*
	HR (95% CI)		HR	
Age (<65 vs ≥65)	1.65 (0.86-3.17)	.120	–	–
Gender (male vs female)	0.81 (0.34-1.94)	.640	–	–
ECOG-PS (0 vs >1)	1.05 (0.55-1.98)	.880	–	–
Etiology (hepatitis vs others)	1.02 (0.54-1.90)	.950	–	–
AFP (<200 vs ≥200 ng/mL)	2.54 (1.30-4.93)	**.006**	1.97 (1.01-3.86)	**.049**
Child–Pugh class (A vs B)	2.91 (1.48-5.70)	**.002**	1.60 (0.75-3.41)	.218
Extrahepatic spread (no vs yes)	0.51 (0.27-0.97)	**.040**	0.41 (0.20-0.82)	**.012**
PNI (<47.6 vs ≥47.6)	0.27 (0.10-0.71)	**.008**	0.24 (0.09-0.65)	**.005**

Bold values indicate statistical significance.

AFP, alpha-fetoprotein; ECOG-PS, Eastern Cooperative Oncology Group-Performance Status; HR, hazard ratio; PNI, prognostic nutritional index;

## Data Availability

The datasets used and/or analyzed during the current study are available from the corresponding author on reasonable request.
